# Do antibiotic residues in soils play a role in amplification and transmission of antibiotic resistant bacteria in cattle populations?

**DOI:** 10.3389/fmicb.2013.00193

**Published:** 2013-07-11

**Authors:** Douglas R. Call, Louise Matthews, Murugan Subbiah, Jinxin Liu

**Affiliations:** ^1^Paul G. Allen School for Global Animal Health, Washington State University, PullmanWA, USA; ^2^Department of Veterinary Microbiology and Pathology, Washington State University, PullmanWA, USA; ^3^Institute of Biodiversity, Animal Health and Comparative Medicine, University of Glasgow, GlasgowScotland, UK; ^4^Department of Veterinary Integrative Biosciences, Texas A&M University, College StationTX, USA

**Keywords:** ceftiofur, cephalosporin, antibiotic resistance, environmental selection

## Abstract

When we consider factors that contribute to the emergence, amplification, and persistence of antibiotic resistant bacteria, the conventional assumption is that antibiotic use is the primary driver in these processes and that selection occurs primarily in the patient or animal. Evidence suggests that this may not always be the case. Experimental trials show that parenteral administration of a third-generation cephalosporin (ceftiofur) in cattle has limited or short-term effects on the prevalence of ceftiofur-resistant bacteria in the gastrointestinal tract. While this response may be sufficient to explain a pattern of widespread resistance to cephalosporins, approximately two-thirds of ceftiofur metabolites are excreted in the urine raising the possibility that environmental selection plays an important additive role in the amplification and maintenance of antibiotic resistant *E. coli* on farms. Consequently, we present a rationale for an environmental selection hypothesis whereby excreted antibiotic residues such as ceftiofur are a significant contributor to the proliferation of antibiotic resistant bacteria in food animal systems. We also present a mathematical model of our hypothesized system as a guide for designing experiments to test this hypothesis. If supported for antibiotics such as ceftiofur, then there may be new approaches to combat the proliferation of antibiotic resistance beyond the prudent use mantra.

## Introduction

Since first being introduced in the 1940's, antibiotics have improved or saved the lives of countless millions of people either directly through disease prevention and treatment, or indirectly by enabling greater food production capacity. Unfortunately, antibiotic use of any kind invariably selects for the emergence, amplification, and persistence of resistant bacteria. The evolutionary dynamics of antibiotic resistance differ depending on how different resistance traits function. For example, when antibiotic resistance involves active efflux or enzymatic degradation pathways, we might expect longer periods to elapse between the introduction of a new antibiotic and adaptation of resistance mechanisms. Of course, naturally produced antibiotics have been selecting for emergence of resistance mechanisms as long as antibiotics have existed in nature (Allen et al., 2010). Consequently, if resistance mechanisms exist, these toolboxes will eventually be co-opted by pathogens. In contrast, antibiotics such as rifamycins, quinolones, and fluoroquinolones bind specific proteins where simple chromosomal mutations are sufficient to alter the binding sites and produce clinically relevant resistance. Acquisition of these mutations can occur during antibiotic exposure, and resistant organisms can subsequently sweep through populations that experience drug selection pressure (Humphrey et al., 2005). Despite the relative ease with which these chromosomal mutations can arise for quinolones, and by extension fluoroquinolones, more complex resistance mechanisms of resistance have already been documented in the field (Xia et al., 2010).

There are several strategies to address the challenge of antibiotic resistance with introduction of new antibiotics being an important avenue in the past 50 years. Aside from the multitude of challenges that now limit development of new antibiotic products (Spellberg et al., 2008), there are fewer completely novel antibiotics being developed and thus we can expect less time to pass between introduction of new products and emergence of widespread antibiotic resistance. For example, a recent review identified 20 new antibiotics that are currently working through product development pipelines (Butler and Cooper, 2011). Of these, 9 are synthetic compounds and 8 of these are quinolones for which we can predict relatively rapid emergence of resistance. Consequently, it is unlikely that we will stay ahead of this problem in the future through new drug development alone.

“Prudent use” is an important policy-based strategy to combat the growing antibiotic resistance challenge by ensuring that antibiotics are only used when they are actually needed, that the most appropriate antibiotic is used for a given disease agent, and that exacting dosage guidelines are followed (Gyssens, 2011). While emergence of antibiotic resistance is inevitable, prudent use practices are likely to limit the overall level of drug selection pressure and consequently limit the equilibrium prevalence of resistant pathogens (Austin et al., 1999). Greater attention to biosecurity and infection prevention measures, use of rapid diagnostics, and use of effective vaccines and probiotics should also reduce disease incidence and thus reduce demand for antibiotics.

Attention to prudent use invariably brings scrutiny to antibiotic use practices in food animal production. From a “mass-action” perspective, more antibiotics are used in food animal production than in human medicine and consequently this sector may contribute disproportionately to development of antibiotic resistance (Sarmah et al., 2006). In the U.S. an estimated 3.3 million kg of antibiotics were sold for human use in 2010 (FDA, 2012b) while 12.2 million kg (FDA, 2011) were sold for use in food animal applications. There are a number of reasons why we should be cautious about directly comparing these numbers (FDA, 2012a), but it is important to note that monensins account for 28.9% of the total sales for use in food animals. These ionophores are entirely unique from antibiotics that are used in people and they function by transporting ions across bacterial cell membranes thereby disrupting ion gradients and killing susceptible bacteria (Callaway et al., 2003). Resistance to monensins has been suggested, but it is not clear if this is a function of intrinsically resistant populations dominating in a community exposed to monensins, or due to emergence of novel resistance mechanisms. To date, no genetically-encoded, horizontally transmissible resistance traits have been described for monensins (Callaway et al., 2003) and there is no recognized means by which use of monensins contribute to selection for other antibiotic resistance traits in pathogenic or commensal bacteria.

The largest component of antibiotic sales for food animals includes oxytetracycline and chlortetracycline (42.2%) (FDA, 2011) and these are mostly used as in-feed additives to promote animal health and growth. It could be argued that because tetracyclines make-up a relatively small percentage of demand in human medicine (3.9%) (FDA, 2012a), resistance to these drugs has a limited potential to impact human health and thus these compounds are a “good” choice relative to other options that might be employed. An important caveat to this conclusion is that tetracycline resistance is commonly associated with multidrug resistant bacteria (FDA, 2012c) and thus selection that favors tetracycline resistance will also co-select for other genetically-linked antibiotic resistance traits. Co-selection of this nature has been described for other antibiotics and toxins such as heavy metals (Stepanauskas et al., 2006; Tremblay et al., 2012).

The therapeutic use of veterinary antibiotics, while representing a lower total mass of antibiotics, is also heavily scrutinized as a contributor to the antibiotic resistance crisis. In the U.S. this is probably best exemplified by fluoroquinolone use in poultry production. Flock-wide treatment (metaphylactic) with an antibiotic such as enrofloxacin (veterinary antibiotic) exerts selective pressure for resistance to the antibiotic ciprofloxacin (human antibiotic) in *Campylobacter jejuni*, which is a non-target bacterium in this application. Because simple chromosomal mutations quickly lead to ciprofloxacin resistance and because poultry is a major reservoir for transmission of *C. jejuni* to people, fluoroquinolones are no longer approved for use as a metaphylactic treatment via poultry water (FDA, 2005). Unfortunately, despite this withdrawal in 2005, the prevalence of ciprofloxacin resistant *C. jejuni* has remained >20% by 2010 for poultry and human clinical isolates alike in the U.S. (FDA, 2012c). It is unclear if this persistence is due to alternative exposure routes or due to resistant strains disseminating via food and travel. Some mutations that convey resistance to ciprofloxacin might also be fitness neutral or they might convey a fitness advantage to the bacteria. If so, the prevalence of ciprofloxacin resistant *C. jejuni* is unlikely to decrease significantly even after cessation of enrofloxacin use in poultry medicine (Luo et al., 2005).

Another important therapeutic antibiotic in production medicine is ceftiofur. This third-generation cephalosporin is widely used as an injectable antibiotic to treat respiratory infections, metritis, and pododermatitis in cattle, but it has also been used in swine, small ruminants, and poultry. Ceftiofur gained rapid acceptance since the 1990's, particularly in the dairy sector (Zwald et al., 2004; Sawant et al., 2005; Sarmah et al., 2006; Saini et al., 2012) because it is a very effective antibiotic with no withholding time for milk production. Nevertheless, increasing resistance to third-generation cephalosporins in *Salmonella* recently led the US Food and Drug Administration to adopt new rules banning non-therapeutic and extra-label uses of this important veterinary antibiotic (FDA, 2012d). There is debate as to whether or not these regulatory changes will have a significant impact on use practices (Wittum, 2012), and as we outline below these changes are likely to have little impact on the amplification of resistant enteric bacteria if amplification of resistant populations is primarily driven by excreted ceftiofur metabolites in the environment.

## Environmental fate of antibiotics

Ceftiofur poses an interesting conundrum because while resistance is prevalent in *E. coli* and *Salmonella* in the U.S. (Winokur et al., 2000; Donaldson et al., 2006; Daniels et al., 2007, 2009; Sawant et al., 2007; Heider et al., 2009; Lindsey et al., 2009), empirical studies show either no treatment effect (Singer et al., 2008; Daniels et al., 2009; Mann et al., 2011) or transient and relatively short-term amplification of resistant populations (Jiang et al., 2006; Lowrance et al., 2007) after administration of ceftiofur. Lowrance et al. (2007) provided the most convincing evidence that selection occurs in the gastrointestinal tract where there was a proportional increase in the number of ceftiofur-resistant *E. coli* following 1, 2, or 3 days administration of a ceftiofur crystalline-free acid product. This study included 61 feedlot steers and resistant *E. coli* were found between 1 and 2 log higher densities compared with control animals, and the effects lasted ~2 weeks. Two-weeks might be considered a relatively short-term impact because it is also consistent with a failure of survey studies to find a relationship between the percentage of cows with ceftiofur-resistant *E. coli* and the percentage of cows treated at the herd level (Tragesser et al., 2006; Daniels et al., 2009; Heider et al., 2009). Jiang et al. (2006) reported an ~0.5 log increase in the number of ceftiofur-resistant *E. coli* immediately following treatment of three calves with a ceftiofur hydrochloride product. In contrast, Mann et al. (2011) reported no significant effects on the proportion of ceftiofur-resistant *E. coli* after administration of a ceftiofur hydrochloride product (*n* = 42 animals). Singer et al. (2008) also reported no change in the number of ceftiofur resistant *E. coli* (*n* = 10 animals). These latter studies did report a significant decrease in total *E. coli* following ceftiofur administration. Thus, if ceftiofur administration impacts the intestinal flora, amplification of ceftiofur-resistant *E. coli* populations is variable with outcomes ranging from no detectable effect to 1–2 log increases probably depending, in part, on the dose (e.g., 2.2 mg/kg vs. 6.6 mg/kg) and number of sequential treatments.

Given the inconsistent and relatively short-term effects of ceftiofur treatment on *E. coli* populations in the gut, this raises the question of whether selection *in vivo* represents the entire story. For example, resistant strains may be originating from other sources and are being transported to cattle via feed or water supplies. Until recently (FDA, 2012d), ceftiofur could be used as an intramammary prophylactic treatment and this practice has been associated with an increased prevalence of ceftiofur-resistant fecal coliform bacteria (Mollenkopf et al., 2010). The authors of this latter study noted that we normally would not expect local administration of an antibiotic in cow udder to affect enteric flora, but the authors proposed that amplification of resistant bacteria and subsequent shedding into the environment and contact transmission could explain this result. In a similar manner, resistant populations of bacteria such as *E. coli* could be exposed to antibiotic residues in urine and feces resulting in selective amplification of the resistant populations in the environment with a subsequent increased risk of contact-dependent transmission and colonization of livestock with resistant *E. coli*.

For an environmental selection process to be important a sufficient concentration of biologically active compound must be present for a sufficient duration to impact the bacterial community. Furthermore, animals exposed to higher densities of resistant bacteria must be at a greater risk of contact transmission and, ultimately, the resistant bacteria must colonize new hosts. Environmental surveillance studies have typically reported that the concentration of antibiotic residues in soil and water varies between parts per trillion (ppt) and parts per billion (ppb) (Winokur et al., 2000; Tolls, 2001; Koplin et al., 2002; Thiele-Bruhn, 2003; Sawant et al., 2007; Ji et al., 2012); values typically well below the concentration needed (ppm) to demonstrate effects from antibiotics *in vitro*. There is speculation that bacteria could be impacted by additive or synergistic effects or that non-target organisms could be impacted at very low concentrations, but with the exception of possible effects on algae there is little evidence that ppt and ppb concentrations of antibiotics have any impact in the environment (Ji et al., 2012).

**Figure 1:**
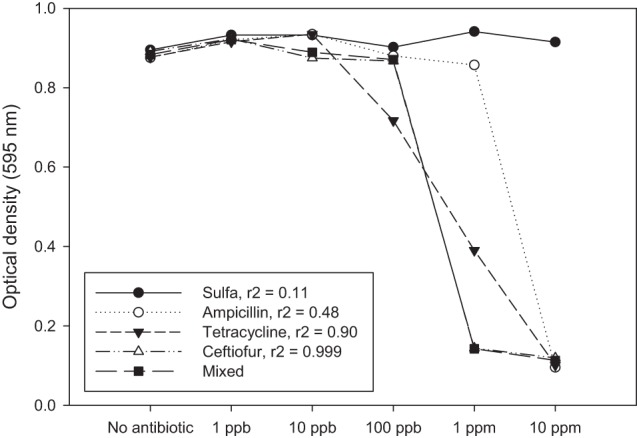
**Antibiotic residues only affect bacteria from a fitness standpoint when the concentration of residue approaches a minimum inhibitory concentration (MIC).** MIC values are typically > 1 ppm, whereas most environmental residue reported in the literature are found at ppb or ppt. These might be significant if there is an additive or synergistic effect attributed to exposure to multiple low-dose antibiotic residues. In this example, single antibiotics and a mixture of four antibiotics at the same concentration were tested against a sensitive strain of *E. coli* (K-12). The response to the mixture is most closely associated with ceftiofur (*r*^2^ = 0.999). If there was an additive or synergistic effect on fitness we would expect to see the mixed antibiotic (solid square) as having the lowest optical density at 100 ppb, which is not the case in this experiment. Each data point represents the optical density after 24 h growth in LB as measured using a Bioscreen plate reader. Average for three independent replicates is shown.

Another way to address this question is to mix combinations of antibiotics at low doses and determine if there is an additive or synergistic effect. Using a simple experiment we show here that there is no evidence for inhibition of bacterial growth *in vitro* until the antibiotics being used reach a concentration where we should expect inhibition of a sensitive strain (i.e., ppm) (Figure 1). There is also reason to expect that any subtle additive or synergistic effects on fitness will be even less prominent in the environment where bacteria typically reside within biofilms that are recalcitrant to antibiotics (Lewis, 2007) and where antibiotic bioavailability may be compromised (Subbiah et al., 2011).

Studies of environmental residues typically measure antibiotics using analytic tools such as ELISA, HPLC, and mass spectrometry (Koplin et al., 2002; Sczesny et al., 2003; Thiele-Bruhn, 2003; Kumar et al., 2004; Aga et al., 2005; Berendsen et al., 2012); methods that can be exquisitely well-suited for detecting minimal concentrations of antibiotics, but these methods do not tell us if the antibiotic residues being detected are biologically available. To be biologically available, antibiotics need to be physically available to interact with target cells. Antibiotics such as tetracyclines, however, are known to rapidly adsorb to clay surfaces (Tolls, 2001) rendering them unavailable to exert biological effects in soils. Subbiah et al. (2011) explored this process further by mixing high concentrations of antibiotics (200 ppm) with soil slurries that differed in clay content, pH and other properties. After mixing, supernatant was recovered, filter sterilized, and added to a broth culture of an antibiotic sensitive *E. coli* strain. If the strain grew, this indicated that there was an insufficient concentration of antibiotic in the liquid phase to have any impact on the test strain because the antibiotic had adsorbed to the soil materials. Using this assay, tetracycline, neomycin, and ciprofloxacin had no effect on bacterial growth after mixing with sand-loam and silt-loam soils; tetracycline, ciprofloxacin, and two sulfonamide antibiotics retained at least partial activity in sand. Even when antibiotic sensitive *E. coli* were added to a viscous soil slurry with adsorbed tetracycline, there was no evidence of an impact on fitness (Subbiah et al., 2011). Florfenicol and β-lactams, however, were mostly available in the liquid phase and these antibiotics still affected the test bacteria. These results are mostly consistent with what could be predicted from the organic normalized dissociation constants (*K*_oc_) for these compounds (Table 1). *K*_oc_ provides an imperfect prediction of how tightly antibiotics adsorb to soil (Tolls, 2001), but at the extremes tetracycline adsorbs very tightly (*K*_oc_ > 420,999) while florfenicol and sulfadiazine adsorb very weakly (*K*_oc_ < 38 and 61, respectively). Ceftiofur is considered slightly mobile by this measure (Table 1), but ceftiofur may exist as an anion in soils and this would increase mobility thus biological availability.

**Table 1:** **Sorption coefficients and expected mobility for select antibiotics in the environment**.

**Antibiotic**	**Class**	***K*_oc_^1^**	**Mobility^2^**
Trimethoprim	2,4-diamino pyrimidine	Median 2589	Slightly mobile
Ampicillin	β-lactam	2728	Slightly mobile
Ceftiofur	β-lactam	3700	Slightly mobile
Penicillin G	β-lactam	N.A.	Slightly mobile^3^
Neomycin	Aminoglycoside	N.A.	Non-mobile?
Ciprofloxacin	Fluoroquinolone	61,000	Non-mobile
Enrofloxacin	Fluoroquinolone	Median 99,975	Non-mobile
Lincomycin	Macrolide	111	Moderately
			mobile
Tylosin	Macrolide	Median 1264	Slightly mobile
Florfenicol	Amphenicols	38	Mobile
Chlortetracycline	Tetracycline	Median 400,522	Non-mobile
Oxytetracycline	Tetracycline	Median 47,932	Non-mobile
Tetracycline	Tetracycline	Median 420,999	Non-mobile
Sulfadiazine	Sulphonamide	61	Mobile

1 K_oc_, estimated organic carbon normalized sorption coefficient (L kg^−1^); values collated from Sarmah et al. (2006); Pavlovic et al. (2007), and Metcalfe et al. (2009), and from the Veterinary Substances Database (VSDB, http://sitem.herts.ac.uk/aeru/vsdb/index.htm).

2 Mobility classification, Very mobile (K_oc_ < 15), Mobile (K_oc_ = 15–74), Moderately mobile (K_oc_ = 75–499), Slightly mobile (K_oc_ = 500–4000), Non-mobile (K_oc_ > 4000) (Pope et al., 2009).

3 Assumed to be similar to other β-lactams.

In a subsequent study Subbiah et al. (2012) focused on the fate of ceftiofur metabolites in soils. When injected into cattle, ceftiofur is rapidly metabolized with most of the metabolite being biologically active desfuroylceftiofur (Hornish and Kotarski, 2002). Jaglan et al. (1989) estimated that 70 and 30% of the metabolites are then excreted through urine and feces, respectively. Ceftiofur metabolites are mostly excreted within 24 h and the concentration varies between ~ 7 and 160 ppm in the urine (El-Gendy et al., 2007; Subbiah et al., 2012); values well within the range needed to affect sensitive bacteria *in vitro*. When urine containing ceftiofur metabolites was added to a soil:feces microcosm (25:1), inhibition from the antibiotic was evident for 3 days at room temperature. Biological degradation appeared to explain the loss of the residues in part because at 4°C the residues were biologically available up to 3 weeks from the start of the experiment. Importantly, exposure to urine containing ceftiofur metabolites was sufficient to produce a log greater increase in growth for a resistant strain of *E. coli* compared to the same strain that was exposed to urine without ceftiofur metabolites. Exposure to ceftiofur metabolites in urine also resulted in > 2 month longer retention of the resistant *E. coli* strain compared to the same strain in control microcosms.

In the case of ceftiofur, biologically active metabolites are excreted at a sufficient concentration that remains available to affect sensitive bacteria in soil. Clearly, the distribution of excreted residues will be very heterogeneous in a cattle herd depending on where treated animals urinate and how materials are disturbed and moved. Furthermore, for this environmental selection scenario to be feasible the amplified populations of resistant *E. coli* on the soil surface must present a greater risk of transmission back to naïve animals compared with conditions where resistant populations undergo no selective amplification. Subbiah et al. (2012) used a controlled experiment to show that bedding contaminated with a ceftiofur resistant strain of *E. coli* is sufficient to colonize naïve calves by contact transmission alone, although a wider range of concentrations and environmental conditions need to be explored to validate this component of an environmental selection scenario.

Lowrance et al. (2007) demonstrated a clear *in vivo* selection effect from ceftiofur that was dependent on the number injections of a ceftiofur product (a high dose per injection, 6.6 mg/kg compared to most studies, 2.2 mg/kg). Importantly, the untreated control animals in this study mingled freely with the treated animals and yet did not show a strong response to the presence of the treated animals. The control animals were clearly colonized and apparently they shed a higher concentration at the study outset (app. 3.5 Log_10_/g feces) compared to 2 weeks later (app. 3.0 Log_10_/g feces), but cause and effect for this response is confounded, in part, by prior colonization and there was no isolated control group for comparison. We further surmise that a number of factors enhance or diminish an environmental effect such as the season, floor substrate, dose delivered, number of injections, density of livestock, waste management practices, animal age, and animal behavior. As a simple example, Lowrance et al. (2007) studied beef cattle (steers) that probably bed down in contaminated substrates less frequently than young calves thereby limiting an important transmission pathway. If environmental selection is an important factor in the amplification and persistence of antibiotic resistant bacteria, and if this process only makes a significant contribution under certain environmental conditions, then it is critical to determine which factors can be modified to limit selection and transmission as much as possible.

## Environmental selection hypothesis

Volkova et al. (2012) provided a comprehensive mathematical model of the dynamics of ceftiofur-sensitive and resistant commensal *E. coli* in the cow large intestine. This model considered population size and growth rate *in vivo*, fitness cost of plasmid carriage by resistant bacteria, and the effect of ceftiofur metabolites in the intestine (entering via bile salts). Volkova and colleagues focused on the *in vivo* selection compartment, but their model included an “in flow” component from the environment through which resistant and sensitive bacteria enter the cow. They concluded, in part, that the rate of replacement *E. coli* acquired through ingestion is an important factor when these strains are ceftiofur resistant. Volkova et al. (2013) explicitly addressed approaches for controlling plasmid-mediated resistance in enteric commensal bacteria and their model also included multiple pathways by which resistant bacteria can be ingested. Our proposed model focuses on selection in the environment based, in part, on the fact that approximately two-thirds of biologically active metabolite after ceftiofur administration is excreted in the urine and thus may be a significant factor leading to amplification of resistant bacteria that subsequently enter the Volkova model via the “in flow” parameter. Notably, neither Volkova et al. (2012) nor Volkova et al. (2013) considered the effects of excreted antibiotic in their scenarios. From a “mass action” perspective, selection in the environment may play a more significant role in this process. Nonetheless, it is possible that a more accurate model will be a hybrid of both the *in vivo* and *ex vivo* selection compartments.

**Figure 2:**
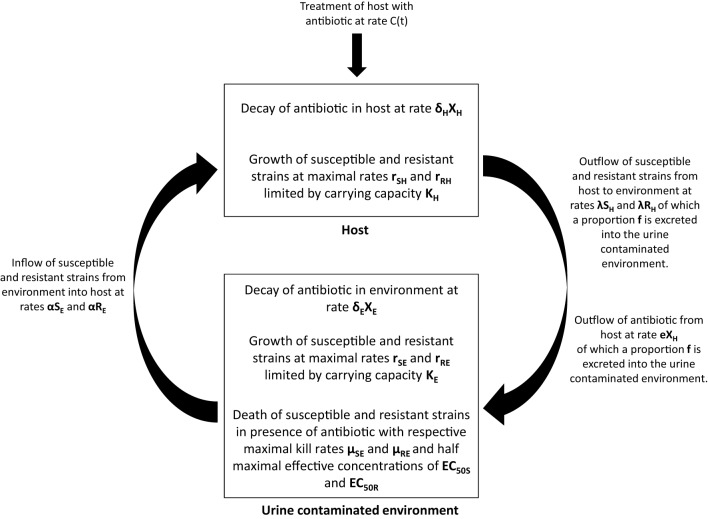
**Graphical depiction of the environmental selection hypothesis by which excreted antibiotic metabolites selectively amplify resistant bacteria in the environment.** Amplification of resistant bacteria subsequently increases the probability of transmission back to the host. See text for details about model parameters.

We hypothesize that excreted ceftiofur contributes to amplification and persistence of resistant enteric bacteria in soils, and consequently use of these antibiotics increases the risk that resistant bacteria will be transmitted back to cattle (Figure 2). While our focus is centered on ceftiofur in these discussions, if the environmental selection hypothesis is valid then it likely applies to other excreted antibiotics that remain biologically available after contact with the ground substrate (Subbiah et al., 2011). A mathematical representation of this hypothesis can be formulated as follows:

$$\begin{array}{l} \frac{dS_{H}}{dt}=\alpha S_{E}+r_{S_{H}}(1-\frac{\left(S_{H}+R_{H}\right)}{K_{H}})S_{H}-\lambda S_{H}\\ \frac{dR_{H}}{dt}=\alpha R_{E}+r_{R_{H}}(1-\frac{\left(S_{H}+R_{H}\right)}{K_{H}})R_{H}-\lambda R_{H}\\ \frac{dS_{E}}{dt}=\lambda fS_{H}+r_{S_{E}}(1-\frac{\left(S_{E}+R_{E}\right)}{K_{E}})S_{E}-\frac{\mu_{SE}S_{E}X_{E}^{\gamma }}{{\left(EC_{50S}\right)}^{\gamma }+X_{E}^{\gamma }}-\alpha S_{E}\\ \frac{dR_{E}}{dt}=\lambda fR_{H}+r_{R_{E}}(1-\frac{\left(S_{E}+R_{E}\right)}{K_{E}})R_{E}-\frac{\mu_{RE}R_{E}X_{E}^{\gamma }}{{\left(EC_{50R}\right)}^{\gamma }+X_{E}^{\gamma }}-\alpha R_{E}\\ \frac{dX_{H}}{dt}=C(t)-\delta_{H}X_{H}-eX_{H}\\ \frac{dX_{E}}{dt}=feX_{H}-\delta_{E}X_{E} \end{array}$$

where *S*_*H*_ and *R*_*H*_ represent susceptible and resistant strains in the host, and *S*_*E*_ and *R*_*E*_ represent susceptible and resistant strains in the urine contaminated environment, respectively. We are initially assuming that there is no selection by ceftiofur in the host, that the host population ingests bacteria from the environment at a rate α and excretes bacteria into the whole environment at rate λ, and that a proportion *f* of bacteria is excreted into areas contaminated with urine.

Within the host, the net replication rate of the strains is given by *r*_*SH*_ and *r*_*RH*_, and the total size of the resistant and susceptible population is limited by a carrying capacity *K*_*H*_. Within the urine contaminated environment, the dynamics of the susceptible and resistant populations are similarly dependent on the ingestion and excretion of strains by the host and by growth that is limited by a carrying capacity *K*_*E*_. If *in vivo* selection proves to be a more significant selection factor than we presume based on the literature, then an *in vivo* selection parameter can be added to the model.

In addition, the dynamics in the urine contaminated environment also include terms that capture antibiotic induced death of the resistant and susceptible strains, where μ_*SE*_ and μ_*RE*_ are the maximal kill rates, *X*_*E*_, the concentration of antibiotic in the environment, γ the Hill coefficient, and *EC*_50*S*_ and *EC*_50*R*_ the half maximal effective concentration for the susceptible and resistant strains, respectively (Vinks, 2002). The concentration of antibiotic in the host is determined by a treatment rate, *C(t)*, a decay rate in the host, δ_*H*_, and an excretion rate into the environment, *e*.

To test the environmental selection hypothesis for ceftiofur we propose to compare the rates of acquisition of resistance among calves in groups including both ceftiofur treated and untreated animals. The calves would initially be presumptively free of ceftiofur-resistant *E. coli* and they would be introduced into an environment where ceftiofur resistant *E. coli* are present. We propose to then compare acquisition rates in groups that differ in the proportions of treated animals. Our hypothesis predicts a more rapid acquisition of resistant strains in groups with a higher proportion of treated animals because greater excretion of ceftiofur will allow greater environmental selection, but no difference between the treated and untreated animals within a group. Specifically, by monitoring animals for colonization, we can obtain estimates for α*R*_*E*_, the rate of acquisition of resistant strains, and compare these estimates between groups and between treated and untreated animals. Additionally, comparing environmental concentrations of resistant strains between groups with high and low proportions of treated animals, and relating these concentrations to acquisition rates, would enable us to estimate the uptake parameter α .

Volkova et al. (2012) describes the mechanism by which resistance may persist within the host via plasmid mediated transfer, whilst the model of Volkova et al. (2013) additionally captures plasmid-mediated transfer in the environment. Our goal, however, is to specifically assess whether environmental selection of strains in the presence of ceftiofur is a more important source of resistance acquisition than within host selection.

## Conclusion

Antibiotic resistance is an increasing challenge to public health worldwide. Currently, our only tools to combat this challenge include developing new antibiotics and preserving the utility of existing antibiotics as long as possible through prudent use principles. Our work suggests that blocking environmental selection could be another important avenue to combat resistance to some antibiotics that are used in food animal medicine. If environmental selection proves to be a significant contributor to maintenance of antibiotic resistance for drugs like ceftiofur (e.g., florfenicol), then it is likely that management and engineered solutions can be devised to limit this component of the selection problem. Importantly, by finding solutions to address unappreciated components of the selection process such as the one described here, it should be possible to extend the utility of important drugs like ceftiofur for use in food animal production. Doing so benefits animal welfare and public health while helping to maintain lower costs for producing food for the burgeoning human population.

### Conflict of interest statement

The authors declare that the research was conducted in the absence of any commercial or financial relationships that could be construed as a potential conflict of interest.
